# Taurine intake ameliorates lactic acidosis and hyperferritinemia occurring after mRNA SARS-CoV-2 vaccination in a patient with β-thalassemia trait: a case report and review of literature

**DOI:** 10.1186/s13256-026-05844-z

**Published:** 2026-01-28

**Authors:** Anthony M. Kyriakopoulos, Peter A. McCullough, Stephanie Seneff

**Affiliations:** 1 Laboratory of Molecular Biology and Immunology, Department of Pharmacy, University of Patras, 26500 Rio-Patras, Greece; 2 McCullough Foundation, Dallas, TX USA; 3 Computer Science and Artificial Intelligence Laboratory, Massachusetts Institute of Technology, Cambridge, MA 02139 USA

**Keywords:** Case study, SARS-CoV-2 mRNA vaccination injury, Taurine, Hyperferritinemia, Lactic acidosis, Mitochondrial dysfunction

## Abstract

**Background:**

Taurine is a powerful antioxidant necessary for mitochondrial function. Lactic acidosis is a complication encountered in the condition mitochondrial myopathy, encephalopathy, lactic acidosis and stroke-like episodes (MELAS), which can be successfully treated with supplemental taurine. Furthermore, taurine regulates the production of iron-dependent proteins such as ferritin that can act as chelating agents to sequester labile iron.

**Case presentation:**

A 38-year-old Greek male with a β-zero thalassemia trait developed multiple severe symptoms soon after his first and only mRNA (Pfizer) SARS-CoV-2 vaccination that included hematological stress to be a candidate for blood transfusion. Amongst the hematological readings, the patient had lactate levels > 4 mmol/ml, indicating lactic acidosis, and ferritin levels > 820 ng/ml, representing hyperferritinemia. Moreover, the patient has organic acid and plasma metabolite levels in the urine that are indicative of mitochondrial dysfunction. Regular taurine intake (500 mg/day) for years helped the patient control lactate and ferritin levels and avoid more serious clinical decompensation.

**Conclusion:**

Regular taurine intake helps to avoid lactic acidosis and reverse hyperferritinemia after mRNA SARS-CoV-2 vaccination in a patient with β-zero thalassemia trait with no obvious genetic trait linked to mitochondrial myopathy, encephalopathy, lactic acidosis and stroke-like episodes. Taurine seemed to be protective for mitochondria.

## Background

Taurine is a natural non-coding amino acid with diverse antioxidant and cytoprotective effects [1]. Taurine has been found to be effective as a natural therapy against a wide range of diseases and conditions, including congestive heart failure, as well as diseases of the muscle, the central nervous system and the cardiovascular system. Taurine appears to be especially effective to treat mitochondrial myopathy, encephalopathy, lactic acidosis and stroke-like episodes (MELAS) and has also shown potential benefit for diabetes and arthritis [2]. Cells actively take up taurine and store it preferentially in the mitochondria, where it acts both as a regulator of osmosis and as a buffer for the alkaline pH of the mitochondrial matrix. The catalytic activity of three enzymes involved in the beta-oxidation of fatty acids in the mitochondria is optimal at the alkaline pH supported by the taurine buffer [3]. MELAS symptoms begin early in life. MELAS occurs owing to the *MTO1* (h*MTO1*) gene mutation that leads to a defect in the 5-taurinomethyluridine (τm^5^U) modification (taurine’s conjugation with uridine) of human mitochondrial (hmt) transfer RNA (tRNA)^LEU(UUR)^. Defects in taurine’s conjugation result in serious mitochondrial dysfunction and a spectrum of diseases [4].

The objective of this study is to show taurine’s effect on lowering lactate and ferritin levels in a non-MELAS case with mitochondrial dysfunction. MELAS is a rare genetic disease that mainly affects the brain and muscles. Symptoms include muscle weakness, dizziness, and cognitive issues. It is associated with a buildup of lactic acid in the blood. Taurine has been shown to have great potential to ameliorate the symptoms of MELAS [5]. Moreover, a deficiency in taurine can lead to a syndrome that in many ways resembles MELAS without an ongoing genetic defect [6]. Finally, taurine is a universal life-saving guardian molecule to ameliorate and prevent symptoms originating from mitochondrial dysfunction [7, 8].

## Case presentation

The 38-year-old Greek male patient, after a single mRNA (Pfizer) vaccination in October of 2020, among cardiovascular, neuromuscular, and respiratory complications, developed an array of hematological complications. His fetal hemoglobin F (Hb-F) levels increased compared with prior to vaccination, and his hemoglobin blood levels dropped to such an extent that he required blood transfusion. Never in his life, as a β-zero thalassemia trait (heterozygous) carrier, had he experienced a similar condition that required blood transfusion. Nor had this patient ever developed in his life any symptoms resembling MELAS, and therefore a genetic defect in taurine conjugation to tRNA is not the case. Overall, his hematological readings resembled a hematological stress condition.

Autoimmunity due to molecular mimicry cannot be disregarded, since the patient had and still has constipation (probably owing to intestinal microbial dysbiosis) and remarkably elevated levels of SARS-CoV-2 immunoglobulin G (IgG) neutralizing antibodies (11,117.80, normal < 50 AU/ml). Because he was experiencing muscle wasting and other disorders (muscle spasms, pain in joints, and muscle cramps), the patient, on his own initiative, at the time of hemoglobin drop, began taking 500 mg of taurine daily for three consecutive months, as a consequence of reading a bibliography about taurine and skeletal muscle wasting. As commented in his medical history report, the taurine intake was lifesaving for him to withstand post-vaccine side effects. Recent laboratory investigations of the patient’s organic acids in urine and plasma metabolites (namely lactic acid and pyruvate), presented in Table 1, are revealing and indicative for mitochondrial dysfunction. In Table 1, only the altered (increased or decreased) values of certain organic acids in urine are presented that are all indicative for mitochondrial deficiency. All other organic acid levels were normal. Among the abnormal levels, nearly all the Krebs cycle respiratory enzymes seem to produce low levels and sometimes diminished (zero levels) of metabolic byproducts. Moreover, although the lactic acid traced in urine is within normal range (8.3 mmol/mol creatinine, not shown in Table 1), the levels of lactic acid in plasma are well above the high limit, rendering the ratio of lactic acid over pyruvate also well above normal levels. Overall, the Table 1 readings are indicative that the enzymes of the mitochondrial respiratory cycle for some reason are not functioning properly.

**Table 1:** The low levels of organic acids in urine and raised lactic acid in plasma that are indicative for mitochondrial deficiency. Test was done more than 4 years post-vaccination

Organic acids in urine Krebs cycle—respiratory chain	Value (18 December 2024)
2-Ketoglucaric acid Normal range: 41–82 mmol/mol creatinine	20
Succinic acid Normal range: 29–87 mmol/mol creatinine	1.3
Fumaric acid Normal range: 0.2–0.8 mmol/mol creatinine	0.0*
Malic acid Normal range: 0.7–5.3 mmol/mol creatinine	1.0
Aconitic acid Normal range: 2.7–44 mmol/mol creatinine	40
Citric acid Normal range: 120–582 mmol/mol creatinine	74.6
Isocitric acid Normal range: 16–97 mmol/mol creatinine	6.9
Plasma/serum metabolites	
Lactic acid Normal range Fasting: 0.7–0.9 mmol/L After meal: 1.0–1.55 mmol/L	3
Pyruvate Normal range Fasting: 0.04–0.12 mmol/L After meal: 0.08–0.16 mmol/L	0.15
Ratio of lactic acid/pyruvate Normal range: 6–14	20

*Zero result is presented as less than 1.0 mmol/mol of creatinine concentration

Among the noticeable symptoms that were relieved with taurine intake were muscle cramps, spasms and neuromuscular pain. Therefore, and, upon the recommendation of a consulting physician at that time, the patient continued to receive the 500 mg of taurine supplementation per day, until today (that is, for more than 2.5 years). Immediately after the mRNA vaccination, the patient had a positive activated partial thromboplastin time-lupus anticoagulant (PPT-LA) ratio result at 1.22 (normal < 1.22), indicative of clotting deficiency. Soon after the mRNA vaccination and before deciding to take 500 mg of taurine daily, the patient had high serum ferritin levels at 821 ng/ml, and his lactate levels were at 4.2 mmol/ml. Prior to the mRNA vaccination, his ferritin levels were normal, at 250 ng/ml, and his lactate levels were at 1.4 mmol/ml. In addition, these were being examined on a routine basis owing to his β-thalassemia trait. After receiving taurine, the elevated levels of both ferritin and lactate dropped (see Table 2), and they continue to represent hyperlactatemia rather than lactic acidosis (> 4 mml/ml) until today.

**Table 2:** The patient’s ferritin and lactate levels before and after mRNA SARS-CoV-2 vaccination and prior to and during taurine intake

Ferritin and lactate levels pre-mRNA SARS-CoV-2 vaccination (28 September 2020)		Date of mRNA vaccination	Ferritin and lactate levels post-mRNA SARS-CoV-2 vaccination (before taurine intake)	Ferritin and lactate levels Post-mRNA SARS-CoV-2 vaccination (During taurine intake, 500 mg/day)			
Serum ferritin (ng/ml). Adult males normal: 6–323 ng/ml	250	29 October 2020	7 January 2021	14 April 2022	21 April 2022	25 January 2024	7 November 2024
			821	438	581	392	279.50
Plasma Lactate (mmol/ml). Adult normal: < 2 mmol/ml	1.4		7 January 2021	21 April 2022	4 January 2024	25 January 2024	7 November 2024
			4.2	2.2	2.31	2.42	3.2

## Discussion

Recently, we have published the rare case of post mRNA vaccination injury, in a 38-year-old β-zero thalassemia trait male patient that constitutes also the subject patient of this case as well [9]. After the mRNA vaccination, the patient’s total blood iron dropped from 70 μg/dL prior to mRNA vaccination to 56 and 58 μg/dL after mRNA vaccination, indicating iron deficiency. [9]. Moreover, the readings of metabolites presented in Table 1 are suggestive for mitochondrial disease, as encountered in states of neurodegeneration (autism) [10]. The raised Hb-F levels (3.3–3.8) and the hemoglobin drop (11.2 g/L) that the patient experienced after mRNA vaccination were indicative of impaired iron homeostasis and sometimes anemia seen in β-thalassemia [9–11]. When this kind of patient suffers from synchronous inflammatory disorders, as often met during mRNA coronavirus disease 2019 (COVID-19) vaccination injuries, likely owing to a high expression of SARS-CoV-2 spike protein [12, 13], the levels of ferritin can be elevated, as seen in our current patient’s case [14]. Ηyperferritinemia induces systemic inflammation, leading to neutrophil infiltration into inflammatory sites and upregulation of myeloperoxidase, producing the highly reactive metabolite, hypochlorite [15]. Taurine, through its spontaneous reaction with hypochlorite, to produce *N*-chlorotaurine (NCT), can protect from tissue damage due to the release of hypochlorite from neutrophils in an inflammatory state [8]. Moreover, NCT, on its own, exerts strong anti-inflammatory activity [1].

Ηyperferritinemia is a serious clinical condition, since it can deprive the organism of iron, with organic complications. Such patients show hemochromatosis in their blood smears [16, 17]. The patient we describe does not have abnormal blood cell morphology other than that of a regular β-thalassemia trait, indicating that the raised ferritin levels do not correlate with an obvious pathology. The high ferritin levels the patient encountered after mRNA vaccination can be responsible for his muscle defects that were alleviated by taurine intake [18].

Post mRNA vaccination hematological complications resemble those encountered in long COVID-19, including hypoxia, thrombotic events, and platelet aggregation–coagulation defects. Moreover, the recombinant SARS-CoV-2 spike protein central region is found to be highly responsible for hemagglutination events [19, 20]. The patient’s symptoms in our case were indicative of clotting deficiency after mRNA vaccination.

Taurine is described to have antiplatelet activity and to be a potential antithrombotic nutrient [21]. Moreover, taurine potentially enhances iron-dependent protein (myoglobin and ferritin) synthesis and ameliorates (lowers) lactic acid levels owing to muscle wasting in the blood in animal models [22, 23]. Iron-chelating compounds such as taurine can decrease the levels of labile iron [24] and, owing to its highly antioxidant properties, can relieve oxidative stress in various disorders, including neuromuscular diseases that involve skeletal muscle wasting [25].

Taurine has a unique and unusual effect on mitochondrial oxidative phosphorylation related to tRNA efficiency. Taurine-methyl-uridine derivatives are nucleosides in mitochondrial tRNA that have conjugated with taurine at selected uridine residues, and this increases the efficiency in decoding specific codons that are essential for critical enzymes involved in mitochondrial respiration. Genetic defects in the *MT-TL1* gene (the m.3243A > G mutation), that encodes tRNA^LUE (UUR)^ are a common cause of the severe condition, MELAS [26]. However, even when the gene is not defective, taurine deficiency can cause inefficient decoding of the affected mitochondrial codons, which lowers the rate of synthesis of certain proteins critical to mitochondrial complex I [4, 7]. Both *MTO1* and *MT-TL1* gene defects are linked to MELAS [27, 28]. Although it cannot be concluded that the patient suffered from taurine deficiency after the mRNA vaccination, since this would have required a special investigation at that time, the alleviation of muscle symptoms resembling MELAS was remarkable, allowing for personal activities at home.

Following a series of human clinical studies on the use of taurine as a therapeutic agent for MELAS, taurine has been found to be nontoxic at exceedingly high concentrations, sometimes reaching 12 g per day, and it can be taken for years [29]. MELAS involves a spectrum of diseases that have as a common factor mitochondrial dysfunction due to failure of respiratory complex I, decreased production of ATP, and elevated oxidative stress, which leads to exceedingly high levels of lactate in the serum. The latest multicentered phase III clinical trial by Oshawa *et al*. reported that, in some cases, there is a 100% reduction of MELAS symptoms lasting for years with continued taurine supplementation [29]. In this case we described, the patient may not be suffering from genetically linked MELAS, but the readings of Krebs cycle enzyme insufficiency (Table 1) are indicative of mitochondrial respiratory complex dysfunction. Moreover, lactic acidosis occurring in MELAS involves lactate levels in blood over 4 mmol/L. However, a level between 2 and 4 mmol/L, which represents hyperlactatemia, is viewed as a less serious clinical condition for the case we describe. Regardless of MELAS, the patient in our case seems to suffer from an insufficiency of Krebs cycle respiratory enzymes, as shown in Table 1, and taurine, as a strong antioxidant, aided to alleviate symptoms of mitochondrial dysfunction.

## Conclusion

This paper describes an interesting case of a severe vaccine reaction in a patient with a β-zero thalassemia trait that was asymptomatic for hematological disorientation before his single mRNA SARS-CoV-2 vaccine. The empirical administration of taurine was indicated in this patient, due to the metabolic sequelae of hematological deterioration and myopathy. Taurine seems to have helped to reduce the levels of lactate and control ferritin as part of his metabolic stabilization. Further larger evaluation studies may prove taurine’s efficiency to be used therapeutically in patients who do not have MELAS but suffer from lactic acidosis and hyperferritinemia. Moreover, taurine may prove to be a useful and effective nutrient supplementation to protect against long COVID and mRNA vaccine injury syndromes. Nevertheless, taurine, by working as a mitochondrial guardian molecule, helped this patient to partly overcome mitochondrial dysfunction and continue with everyday life activities.

## Data Availability

Specific data for this case can be found in our previous publication, 10.56098/y768gc33.

## Abbreviations

τm^5^U: 5-taurinomethyluridine
IgG: Immunoglobulin G
MELAS: Mitochondrial myopathy, encephalopathy, lactic acidosis and stroke-like episodes
NCT: *N*-chlorotaurine
PPT-LA: Partial thromboplastin time-lupus anticoagulant
tRNA: Transfer RNA

## References

[1] Kim C, Cha YN. Taurine chloramine produced from taurine under inflammation provides anti-inflammatory and cytoprotective effects. Amino Acids. 2014;46(1):89–100. 10.1007/s00726-013-1545-6. 23933994 10.1007/s00726-013-1545-6

[2] Schaffer S, Kim HW. Effects and mechanisms of taurine as a therapeutic agent. Biomol Ther (Seoul). 2018;26(3):225–41. 10.4062/biomolther.2017.251. 29631391 10.4062/biomolther.2017.251 PMC5933890

[3] Hansen SH, Andersen ML, Cornett C, Gradinaru R, Grunnet N. A role for taurine in mitochondrial function. J Biomed Sci. 2010;17(Suppl 1): S23. 10.1186/1423-0127-17-S1-S23. 20804598 10.1186/1423-0127-17-S1-S23 PMC2994382

[4] Suzuki T, Suzuki T, Wada T, Saigo K, Watanabe K. Taurine as a constituent of mitochondrial tRNAs: new insights into the functions of taurine and human mitochondrial diseases. EMBO J. 2002;21(23):6581–9. 10.1093/emboj/cdf656. 12456664 10.1093/emboj/cdf656 PMC136959

[5] Rikimaru M, Ohsawa Y, Wolf A, Nishimaki K, Ichimiya H, Kamimura N, *et al*. Taurine ameliorates impaired the mitochondrial function and prevents stroke-like episodes in patients with MELAS. Intern Med. 2012;51(24):3351–7. 10.2169/INTER-NALMEDICINE.51.7529. 23257519 10.2169/internalmedicine.51.7529

[6] Schaffer SW, Jong CJ, Warner D, Ito T, Azuma J. Taurine deficiency and MELAS are closely related syndromes. Adv Exp Med Biol. 2013;776:153–65. 10.1007/978-1-4614-6093-016. 23392880 10.1007/978-1-4614-6093-0_16

[7] Jong CJ, Sandal P, Schaffer SW. The role of taurine in mitochondria health: more than just an antioxidant. Molecules. 2021;26(16): 4913. 10.3390/molecules26164913. 34443494 10.3390/molecules26164913 PMC8400259

[8] Seneff S, Kyriakopoulos AM. Taurine prevents mitochondrial dysfunction and protects mitochondria from reactive oxygen species and deuterium toxicity. Amino Acids. 2025;57: 6. 10.1007/s00726-024-03440-3. 39789296 10.1007/s00726-024-03440-3 PMC11717795

[9] Kyriakopoulos AM, Seneff S. Pathology deterioration in a pure β-zero thalassemia heterozygote after mRNA COVID-19 vaccination: a case report and literature review. IJVTPR. 2024;3(2):1245–73. 10.56098/y768gc33.

[10] Oh M, Kim SA, Yoo HJ. Higher lactate level and lactate-to-pyruvate ratio in autism spectrum disorder. Exp Neurobiol. 2020;29(4):314–22. 10.5607/en20030. 32921643 10.5607/en20030 PMC7492845

[11] Pinto VM, Forni GL. Management of iron overload in beta-thalassemia patients: clinical practice update based on case series. Int J Mol Sci. 2020;21(22): 8771. 10.3390/ijms21228771. 33233561 10.3390/ijms21228771 PMC7699680

[12] Kyriakopoulos AM, Nigh G, McCullough PA, Seneff S. Clinical rationale for dietary lutein supplementation in long COVID and mRNA vaccine injury syndromes. F1000Res. 2024;13: 191. 10.12688/f1000research.143517.3. 39526116 10.12688/f1000research.143517.3 PMC11549548

[13] Lee S, Lee J, Cho SH, Roh G, Park HJ, Lee YJ, *et al*. Assessing the impact of mRNA vaccination in chronic inflammatory murine model. NPJ Vaccines. 2024;9(1): 34. 10.1038/s41541-024-00825-z. 38360752 10.1038/s41541-024-00825-z PMC10869740

[14] Cullis JO, Fitzsimons EJ, Griffiths WJ, Tsochatzis E, Thomas DW, British Society for Haematology. Investigation and management of a raised serum ferritin. Br J Haematol. 2018;181(3):331–40. 10.1111/bjh.15166. 29672840 10.1111/bjh.15166

[15] Jia J, Wang M, Meng J, Ma Y, Wang Y, Miao N, *et al*. Ferritin triggers neutrophil extracellular trap-mediated cytokine storm through Msr1 contributing to adult-onset Still’s disease pathogenesis. Nat Commun. 2022;13:6804. 10.1038/s41467-022-34560-7. 36357401 10.1038/s41467-022-34560-7 PMC9648446

[16] Sandnes M, Ulvik RJ, Vorland M, Reikvam H. Hyperferritinemia—A clinical overview. J Clin Med. 2021;10(9):2008. 10.3390/jcm10092008. 34067164 10.3390/jcm10092008 PMC8125175

[17] Pretorius E, Vermeulen N, Bester J, du Plooy JL, Gericke GS. The effect of iron overload on red blood cell morphology. Lancet. 2014;383(9918):722. 10.1016/S0140-6736(13)61208-8. 24486189 10.1016/S0140-6736(13)61208-8

[18] Halon-Golabek M, Borkowska A, Herman-Antosiewicz A, Antosiewicz J. Iron metabolism of the skeletal muscle and neurodegeneration. Front Neurosci. 2019;15(13):165. 10.3389/fnins.2019.00165. 10.3389/fnins.2019.00165 PMC6436082 30949015

[19] Yazdani AN, DeMarco N, Patel P, Abdi A, Velpuri P, Agrawal DK, *et al*. Adverse hematological effects of COVID-19 vaccination and pathomechanisms of low acquired immunity in patients with hematological malignancies. Vaccines (Basel). 2023;11(3):662. 10.3390/vaccines11030662. 36992246 10.3390/vaccines11030662 PMC10058097

[20] Boschi C, Scheim DE, Bancod A, Militello M, Bideau ML, Colson P, *et al*. SARS-CoV-2 Spike protein induces hemagglutination: implications for COVID-19 morbidities and therapeutics and for vaccine adverse effects. Int J Mol Sci. 2022;23(24):15480. 10.3390/ijms232415480. 36555121 10.3390/ijms232415480 PMC9779393

[21] Roşca AE, Vlădăreanu AM, Mirica R, Anghel-Timaru CM, Mititelu A, Popescu BO, *et al*. Taurine and its derivatives: analysis of the inhibitory effect on platelet function and their antithrombotic potential. J Clin Med. 2022;11(3):666. 10.3390/jcm11030666. 35160118 10.3390/jcm11030666 PMC8837186

[22] Seidel U, Lüersen K, Huebbe P, Rimbach G. Taurine enhances iron-related proteins and reduces lipid peroxidation in differentiated C2C12 myotubes. Antioxidants (Basel). 2020;9(11):1071. 10.3390/antiox9111071. 33142756 10.3390/antiox9111071 PMC7693586

[23] Manabe S, Kurroda I, Okada K, Morishima M, Okamoto M, Harada N, *et al*. Decreased blood levels of lactic acid and urinary excretion of 3-methylhistidine after exercise by chronic taurine treatment in rats. J Nutr Sci Vitaminol (Tokyo). 2003;49(6):375–80. 10.3177/jnsv.49.375. 14974726 10.3177/jnsv.49.375

[24] Fibach E, Rachmilewitz EA. Iron overload in hematological disorders. Presse Med. 2017;46(12 Pt 2):e296–305. 10.1016/j.lpm.2017.10.007. 29174474 10.1016/j.lpm.2017.10.007

[25] De Luca A, Pierno S, Camerino DC. Taurine: the appeal of a safe amino acid for skeletal muscle disorders. J Transl Med. 2015;13: 243. 10.1186/s12967-015-0610-1. 26208967 10.1186/s12967-015-0610-1 PMC4513970

[26] Moraes CT, Ricci E, Bonilla E, DiMauro S, Schon EA. The mitochondrial tRNA(Leu(UUR)) mutation in mitochondrial encephalomyopathy, lactic acidosis, and strokelike episodes (MELAS): genetic, biochemical, and morphological correlations in skeletal muscle. Am J Hum Genet. 1992;50(5):934–49. 1315123 PMC1682620

[27] Ghezzi D, Baruffini E, Haack TB, Invernizzi F, Melchionda L, Dallabona C, *et al*. Mutations of the mitochondrial-tRNA modifier MTO1 cause hypertrophic cardiomyopathy and lactic acidosis. Am J Hum Genet. 2012;90(6):1079–87. 10.1016/j.ajhg.2012.04.011. 22608499 10.1016/j.ajhg.2012.04.011 PMC3370278

[28] Karicheva OZ, Kolesnikova OA, Schirtz T, Vysokikh MY, Mager-Heckel AM, Lombès A, *et al*. Correction of the consequences of mitochondrial 3243A>G mutation in the MT-TL1 gene causing the MELAS syndrome by tRNA import into mitochondria. Nucleic Acids Res. 2011;39(18):8173–86. 10.1093/nar/gkr546. 21724600 10.1093/nar/gkr546 PMC3185436

[29] Ohsawa Y, Hagiwara H, Nishimatsu SI, Hirakawa A, Kamimura N, Ohtsubo H, *et al*. Taurine supplementation for prevention of stroke-like episodes in MELAS: a multicentre, open-label, 52-week phase III trial. J Neurol Neurosurg Psychiatry. 2019;90(5):529–36. 10.1136/jnnp-2018-317964. 29666206 10.1136/jnnp-2018-317964 PMC6581075

